# Estimated incidence of respiratory hospitalizations attributable to RSV infections across age and socioeconomic groups

**DOI:** 10.1186/s41479-022-00098-x

**Published:** 2022-10-25

**Authors:** Zhe Zheng, Joshua L. Warren, Eugene D. Shapiro, Virginia E. Pitzer, Daniel M. Weinberger

**Affiliations:** 1 Department of Epidemiology of Microbial Diseases and the Public Health Modeling Unit, Yale School of Public Health, Yale University, New Haven, CT USA; 2 Department of Biostatistics and the Public Health Modeling Unit, Yale School of Public Health, Yale University, New Haven, CT USA; 3 Department of Pediatrics, Yale School of Medicine, Yale University, New Haven, CT USA

## Abstract

**Background:**

Surveillance for respiratory syncytial virus (RSV) likely captures just a fraction of the burden of disease. Understanding the burden of hospitalizations and disparities between populations can help to inform upcoming RSV vaccine programs and to improve surveillance.

**Methods:**

We obtained monthly age-, ZIP code- and cause-specific hospitalizations in New York, New Jersey, and Washington from the US State Inpatient Databases (2005–2014). We estimated the incidence of respiratory hospitalizations attributable to RSV by age and by socioeconomic status using regression models. We compared the estimated incidence and the recorded incidence (based on ICD9-CM) of RSV hospitalizations to estimate the under-recorded ratio in different subpopulations.

**Results:**

The estimated annual incidence of respiratory hospitalizations due to RSV was highest among infants < 1 year of age with low socioeconomic status (2800, 95% CrI [2600, 2900] per 100,000 person-years). We also estimated a considerable incidence in older adults (≥ 65 years of age), ranging from 130 to 960 per 100,000 person-years across different socioeconomic strata. The incidence of hospitalization recorded as being due to RSV represented a significant undercount, particularly in adults. Less than 5% of the estimated RSV hospitalizations were captured for those ≥ 65 years of age.

**Conclusions:**

RSV causes a considerable burden of hospitalization in young children and in older adults in the US, with variation by socioeconomic group. Recorded diagnoses substantially underestimate the incidence of hospitalization due to RSV in older adults.

**Supplementary Information:**

The online version contains supplementary material available at 10.1186/s41479-022-00098-x.

## Background

Respiratory syncytial virus (RSV) causes a large burden of disease in infants, young children, and the elderly [1, 2]. Although no vaccine against RSV is currently available, multiple vaccines and extended-duration monoclonal antibodies are in development, and preliminary estimates of their efficacy are promising [3, 4]. To accurately assess the potential impact of interventions against RSV, it is essential to evaluate the health burden of RSV in subpopulations with different demographic and socioeconomic characteristics.

Though the epidemic dynamics of RSV vary even within local geographic areas [5, 6], little is known about how the burden of RSV varies between populations. Previous research has had mixed conclusions about the impact of socioeconomic status (SES) on the risk of RSV infections [7–9]. While some studies found lower rates of RSV infection in higher SES groups, others found no relationship. The incidence of hospitalization for bronchiolitis in infants (often caused by RSV) varies greatly between communities of different socioeconomic levels [10–12].

Rates of hospitalizations recorded as being due to RSV infections can also be influenced by testing and coding practices. RSV-associated hospitalizations are likely under-ascertained using laboratory surveillance data. The decision to conduct a diagnostic test for RSV infection and the sensitivity of the tests can influence the completeness of detection. For instance, compared with children, adults are less likely to be tested for RSV, and the diagnostic tests have lower sensitivity in adults [13, 14].

The goal of this study was to quantify the annual incidence of hospitalizations associated with RSV in different localities and to estimate the degree of under-recording by age group across different socioeconomic backgrounds. To achieve this, we fitted regression models to time series data from comprehensive hospitalization databases from three states in the United States (US) to estimate variations in RSV-attributable incidence of respiratory hospitalizations. Our results can be used to improve RSV surveillance and to inform the potential impacts of different strategies to prevent RSV infections.

## Materials and methods

### Overview

We used hierarchical Bayesian regression models to estimate the incidence of respiratory hospitalizations attributable to RSV infections in different age and SES groups. The model incorporated monthly data on the number of hospitalizations recorded as due to RSV infections (based on ICD-9-CM) while adjusting for influenza infections, seasonality and underlying temporal trends. The goal of these analyses was to link variations in the number of hospitalizations recorded as due to RSV infections with variations in the number of all-cause respiratory hospitalizations [15–18].

### Data sources

Data consisting of individual-level hospital discharges from the states of New York, New Jersey, and Washington from July 2005 to June 2014 were obtained from the State Inpatient Databases of the Healthcare Cost and Utilization Project, maintained by the Agency for Healthcare Research and Quality (purchased through the HCUP Central Distributor) [19]. Variables included age at admission, ZIP code of residence, the recorded ICD-9-CM-diagnoses (multiple fields), and the month and the year of hospital admission. Because the coding practices changed from ICD-9-CM-diagnoses to ICD-10-CM-diagnoses in 2015, we chose to limit the analysis to data through June 2014. The outcome in the regression was the number of hospital discharges for respiratory causes by age, month, and SES classification of ZIP code of residence based on median household income at the ZIP-code level. Respiratory hospitalizations were defined as an ICD-9-CM code in the range 460–519 in any of the diagnosis fields (including hospitalizations with diagnoses of pneumonia, bronchitis, and chronic lower respiratory disease, etc.). We analyzed hospitalizations in 9 age categories (< 1, 1, 2–4, 5–9, 10–19, 20–44, 45–64, 65–84, 85 + years).

Median household income at the ZIP-code level was obtained from the US Census Bureau’s American Community Survey. Our analyses were based on the median household income for 2006–2011. We grouped the ZIP codes into three distinctive SES groups based on the tertile of income. ZIP codes were assigned to the same SES group for the entire period. Population estimates for each ZIP code and age group were obtained from the US Bureau of the Census for the year 2010 [20].

The indicator of RSV infections in all age groups was defined as the monthly RSV-specific hospitalizations count (ICD-9-CM code: 079.6, 466.11, 480.1) among children under two years of age in the same SES group. We used RSV infections among children under two years of age as the marker for RSV infections because previous research has shown detection of RSV in children is more accurate than detection in adults [21, 22]. Similarly, to form a more reliable indicator of influenza infections, we aggregated the influenza-specific hospitalizations count (ICD-9-CM code: 487) across age groups in the same SES group.

Time series of monthly counts were created for each income level and age group based on the date of hospital admission for either RSV infections or influenza infections. Data cleaning was performed with SAS software, version 9.2 (SAS Institute, Cary, North Carolina). Statistical analyses were performed in R v4.0.2.

### Statistical model

We estimated the age- and SES-specific incidence of respiratory hospitalizations attributable to RSV using hierarchical Bayesian regression models. The model had a negative binomial likelihood and identity link. The hierarchical structure provides advantages compared with fitting these models individually by group. The model pools information across all groups leading to a reduction in uncertainty during parameter estimation while still allowing for differences between groups [23]. The identity link ensures that each covariate has an additive, rather than a multiplicative, effect on the outcomes of interest [24, 25]. Monthly and yearly dummy variables were used to adjust for seasonality and temporal trends, respectively. Our model used seasonal dummy variables instead of polynomial time trends or sinusoidal curves because the seasonal dummy variable resulted in significantly improved model fit as measured by deviance information criterion (DIC) (Table S1) [17, 18, 26]. Details on the model structure can be found in the supplementary document with code available via GitHub (https://github.com/weinbergerlab/RSVhospitalizations_USA).

After we fitted the model, the estimated “true” number of hospitalizations attributable to RSV infections for each time point and stratum was estimated by multiplying posterior samples of the age-specific scaling factor by the count of monthly hospitalizations that were recorded as due to RSV infections among children under two years of age in the same SES group. The average annual incidence of hospitalizations attributable to RSV infections was estimated by dividing the sum of the group-specific estimated number of hospitalizations attributable to RSV infections over nine epidemiologic years by the age- and site-specific population. The recording ratios were calculated by dividing the number of recorded ICD-9-CM diagnoses for RSV in each age and SES group by the modeled estimates in the same group. The attributable percent of RSV was calculated by dividing the sum of the estimated number of respiratory hospitalizations attributable to RSV infections by the sum of the observed number of all-cause respiratory hospitalizations in each age and SES group over the entire study period. For each measure, we obtained and summarized samples from the posterior distributions of interest. The incidence was rounded to two significant figures. The recording ratios and attributable percent were rounded to the nearest whole number.

## Results

### Recorded hospitalizations

From July 2005 through June 2014, there were a total of 9,418,390 hospitalizations for respiratory illnesses that contained detailed age and ZIP code information across the three states. Age or ZIP code were missing for 0.1% of the records in the database, and these records were excluded. There were 66,679 hospitalizations recorded as being due to RSV infections among children under two years of age during the study period. State-specific information can be found in the supplementary document (Table S1).

### Estimates of RSV-associated respiratory hospitalizations

**Fig. 1:**
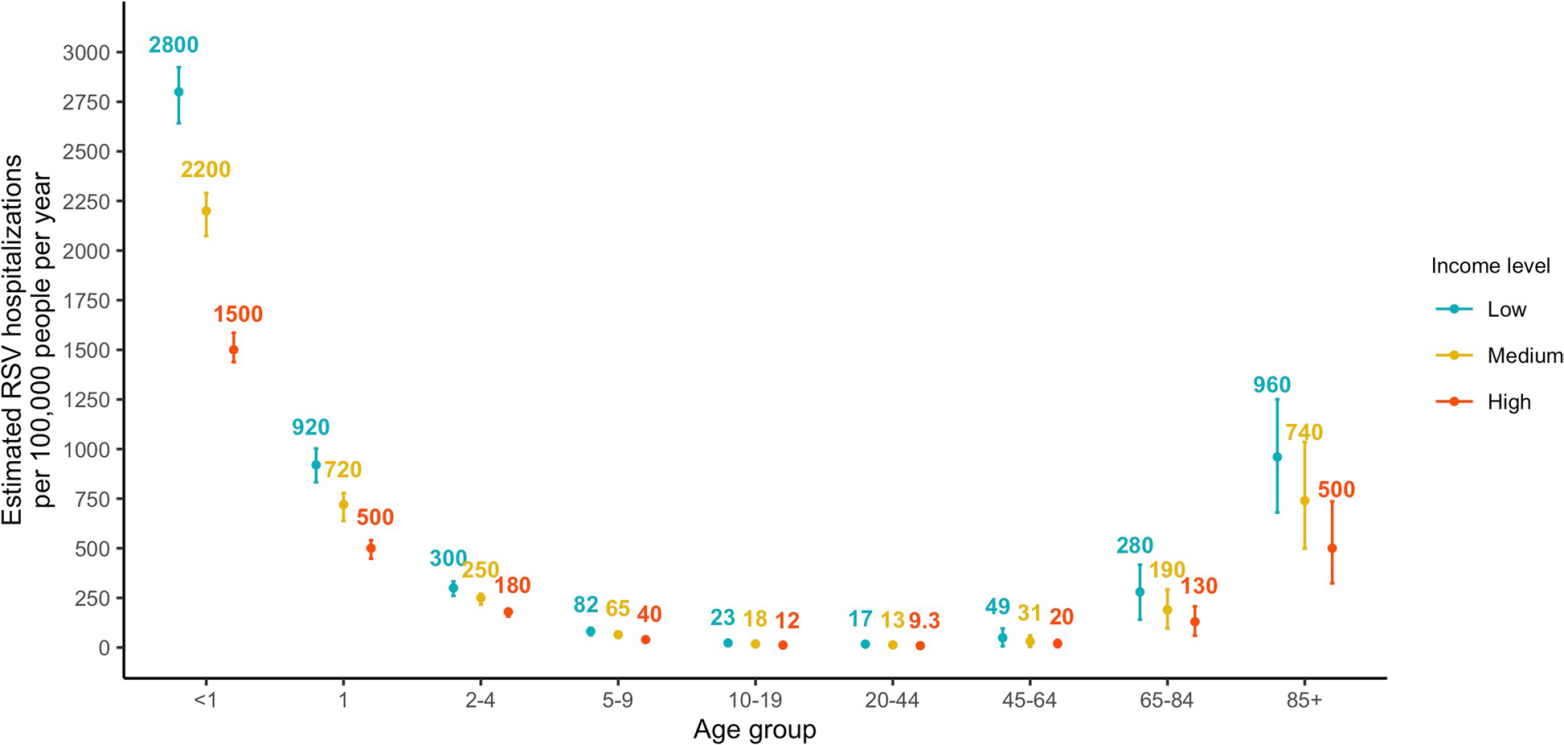
Estimated annual RSV-attributable respiratory hospitalization rates by age and SES group, July 2005—June 2014. The color texts show the mean estimates of RSV-attributable respiratory hospitalization rates per year in each age and SES group. The error bars indicate the 95% credible intervals of the estimated RSV-attributable respiratory hospitalization rates. Color blue, yellow, and red correspond to the estimates in populations from low, medium, and high SES ZIP codes, respectively

The highest estimated annual incidence of hospitalizations attributable to RSV infections was found in infants < 1 year of age (2800, 95% CrI [2600, 2900] per 100,000 person-years), followed by children 1–2 years of age (920, 95% CrI [830, 1000] per 100,000 person-years) and adults ≥ 85 years of age (960, 95% CrI [680, 1300] per 100,000 person-years) (Fig. 1). The estimated annual hospitalization incidence for RSV infections among children in the ZIP codes from the lowest tertile for SES was almost double that of children in the highest SES ZIP codes (Fig. 1). Analyzing the data from each state separately yielded similar results (see Figure S1-S3).

### Gap between recorded and estimated RSV hospitalizations

**Fig. 2:**
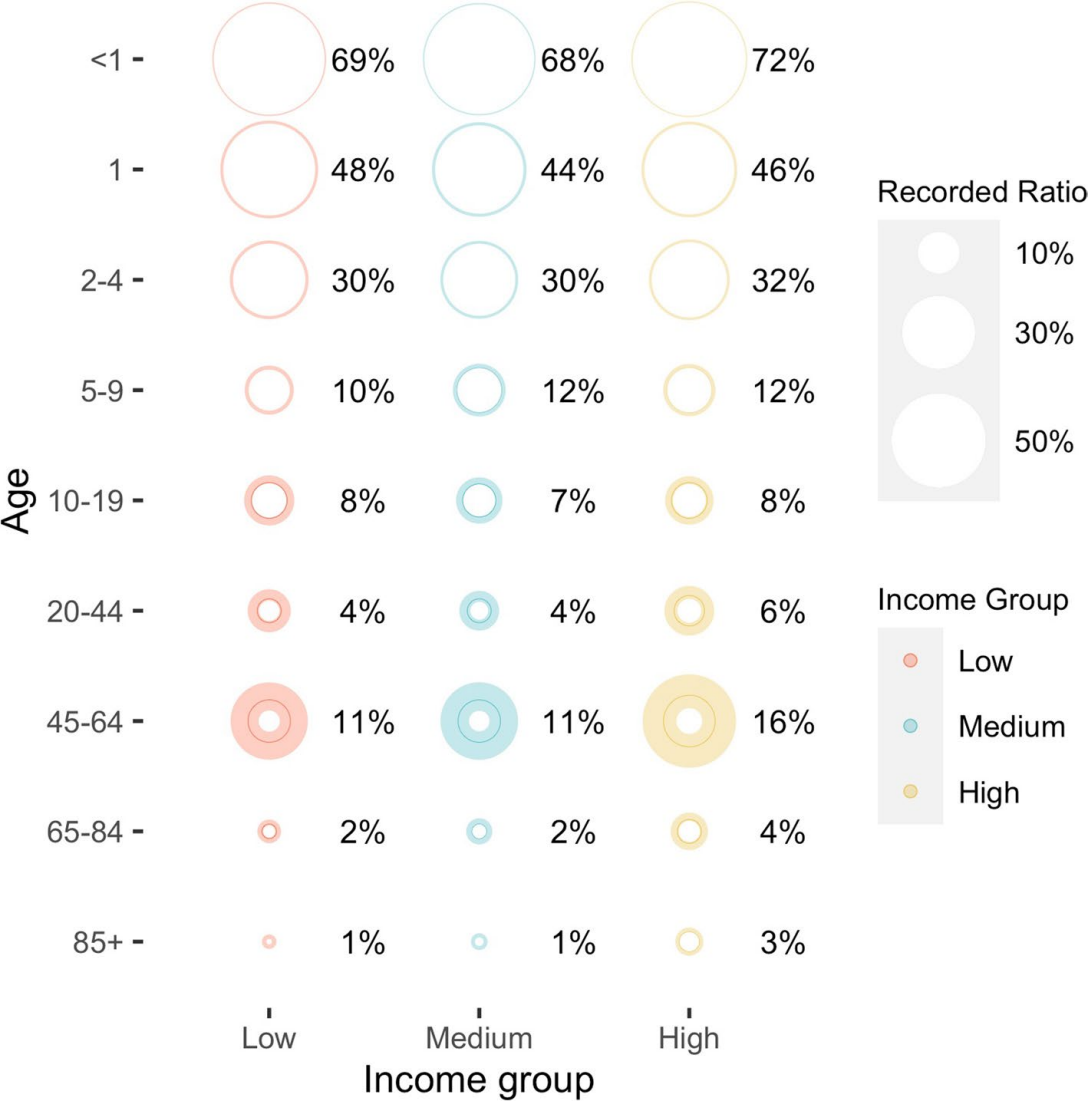
Ratio of the number of annual hospitalizations recorded as being due to RSV and the number of estimated annual RSV hospitalizations, by age and SES group, July 2005—June 2014. The size of the circles represents the estimated proportion of hospitalizations caused by RSV that were recorded. The texts show the mean estimates of the recording ratio in each age and SES group. The shaded areas indicate the 95% credible intervals of the reporting ratio. Color blue, yellow, and red correspond to the estimates in populations from low, medium, and high SES ZIP codes

The estimated proportion of hospitalizations caused by RSV that were recorded as being due to RSV was similar between SES groups and decreased with age (Fig. 2). For example, in the highest SES group, 72% (95% CrI: 69%-76%) of estimated RSV hospitalizations among infants < 1 year of age were recorded as being caused by RSV, whereas only 3% (95% CrI: 2%-5%) of estimated RSV hospitalizations among adults ≥ 85 years of age were recorded as due to RSV.

**Fig. 3:**
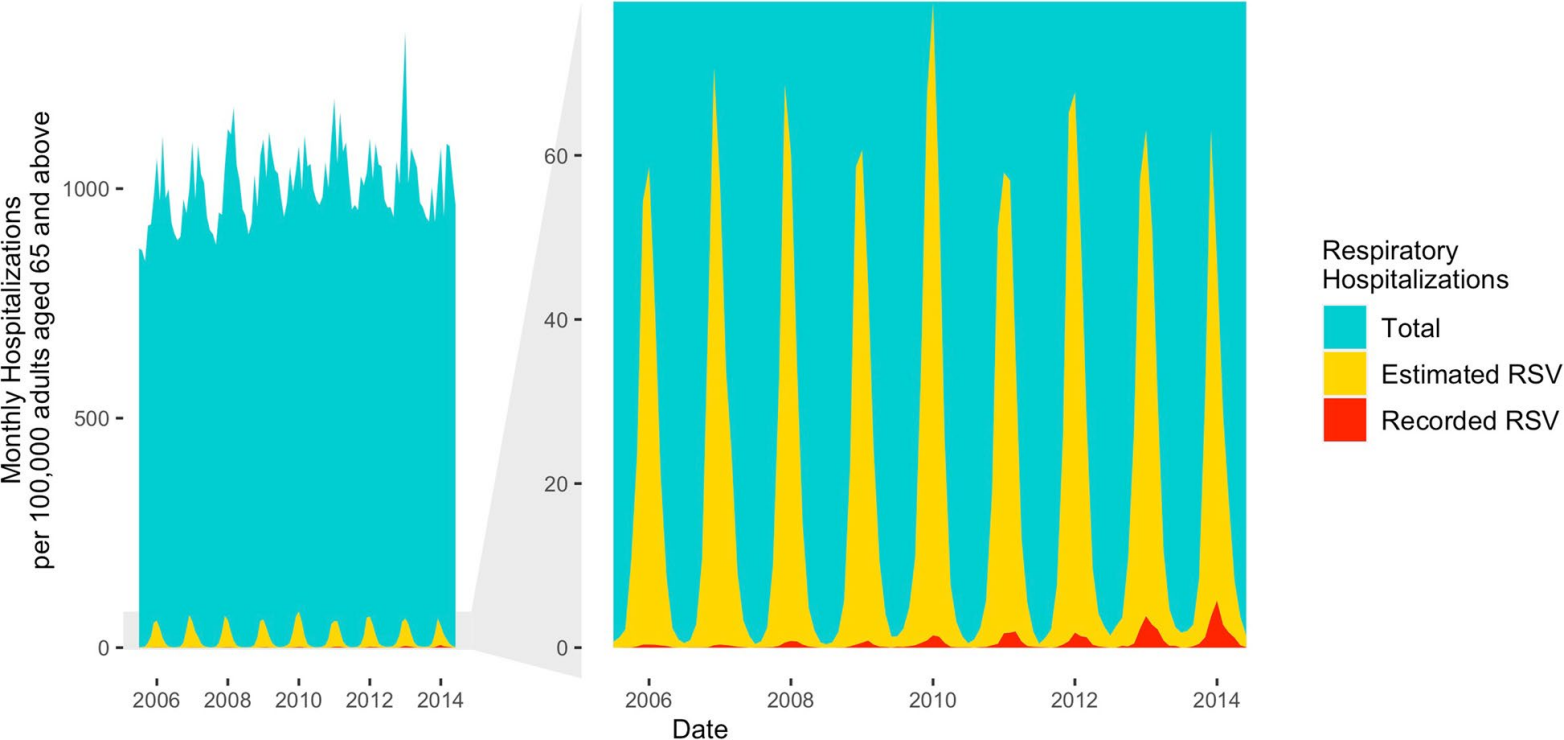
Monthly respiratory hospitalizations among older adults, July 2005—June 2014. The blue area in the left panel shows the total monthly respiratory hospitalization rate among adults aged 65 and above; the yellow area shows the estimated monthly RSV-associated respiratory hospitalizations in the same age group and the red area shows the hospitalizations recorded as RSV each month. The right panel zooms in to show the increasing trend of recorded RSV diagnoses

While most of the estimated hospitalizations attributable to RSV infections among adults ≥ 65 years of age were not recorded as being due to RSV, the rate of recording increased over time (Fig. 3). The estimated recording ratio was 1% (95% CrI: 0%-1%) in January (peak time of RSV epidemics) during the 2005–2006 RSV season and increased to 12% (95% CrI: 9%-18%) in January during the 2013–2014 RSV season.

### Percent of respiratory hospitalizations attributable to RSV

**Fig. 4:**
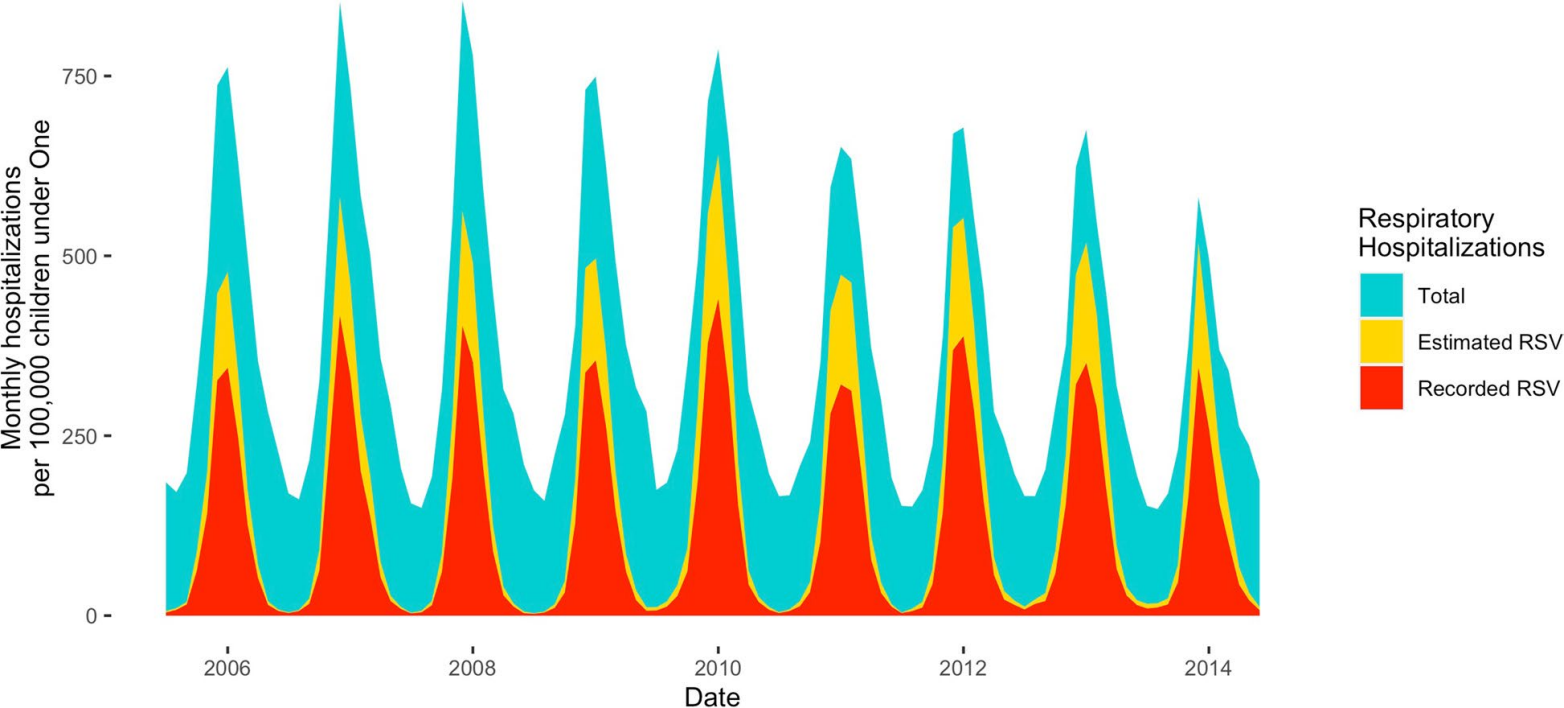
Monthly respiratory hospitalizations in infants < 1 year of age, July 2005—June 2014. The blue area shows the total monthly respiratory hospitalization rate in infants < 1 year of age; the yellow area shows the estimated monthly RSV-associated respiratory hospitalizations in the same age group and the red area shows the hospitalizations recorded as being due to RSV per month

Infections due to RSV were estimated to contribute to a large proportion of hospitalizations for respiratory illnesses in young children (Fig. 4). On average, 45% (95% CrI: 42%-47%) of such hospitalizations were attributable to RSV infection in infants < 1 year of age. This proportion was smaller in older age groups (Table 1). For example, in adults 20–44 years of age, only 1% (95% CrI: 0%-2%) of hospitalizations for respiratory infections were attributable to RSV infection. The attributable percent was similar across SES levels.

**Table 1:** Estimated Percentage of Respiratory Hospitalizations Attributable to RSV infections, by SES and Age Group, 2005–2014

Age (in years)	Low SES		Medium SES		High SES	
	Mean	95% CrI	Mean	95% CrI	Mean	95% CrI
< 1	44%	(42%, 46%)	46%	(44%, 48%)	46%	(44%, 48%)
1	27%	(24%, 29%)	29%	(25%, 31%)	27%	(24%, 29%)
2–4	18%	(16%, 20%)	21%	(18%, 23%)	18%	(16%, 20%)
5–9	9%	(7%, 11%)	11%	(8%, 13%)	9%	(7%, 11%)
10–19	3%	(2%, 4%)	4%	(2%, 5%)	3%	(2%, 4%)
20–44	1%	(0%, 2%)	1%	(0%, 2%)	1%	(0%, 2%)
45–64	1%	(0%, 2%)	1%	(0%, 2%)	1%	(0%, 2%)
65–84	2%	(1%, 3%)	2%	(1%, 3%)	1%	(1%, 2%)
85 +	4%	(3%, 6%)	4%	(2%, 5%)	2%	(1%, 3%)

## Discussion

Understanding the incidence of hospitalizations caused by RSV can help to quantify the potential impacts of upcoming RSV vaccine programs in different subpopulations. In addition, information about the differences in recording ratios between subpopulations may help increase awareness of providers about testing for RSV in older adults and may lead to improvements in RSV surveillance. We estimated a considerable burden of disease in young children (< 5 years of age) and in the elderly (≥ 65 years of age). The estimated incidence of hospitalizations due to RSV was notably higher among patients from low SES ZIP codes. RSV was less likely to be diagnosed in older adults. The percentage of hospitalizations for respiratory illnesses attributable to RSV was highest in infants and lowest among adults 20–64 years of age. With several vaccines and monoclonal antibodies against RSV under active development, these findings can help to inform estimates of the impact of RSV interventions in different populations.

Our age-based estimates for the incidence and attributable percent of hospitalizations due to RSV infections are consistent with previous studies [17, 18, 27]. The statistical models we used in our analysis are an extension of the commonly employed time series models that estimate rates of respiratory-virus-associated hospitalization [15, 28]. By modifying the model structure, our study provides new insights into the variation in RSV-associated respiratory hospitalizations by SES groups. Our estimates of the incidence of hospitalizations attributable to RSV infections among infants < 1 year of age are lower than the average incidence estimates published in the more distant past, but comparable to the averages in recent years [17, 18].

If household income variations are similar across states, we can extrapolate our model-based estimates to other states. After extrapolation, we would expect 160,000 RSV-associated respiratory hospitalizations in children under 5 years of age annually in the US. Our estimate is higher than the estimate of inpatient surveillance by Rha et al. because their inpatient surveillance mainly captured urban populations from November 2015 to June 2016, after the start of an RSV season [29]. We expect 120,000 RSV-associated respiratory hospitalizations in adults ≥ 65 years of age in the US every year. This estimate of annual RSV hospitalizations is similar to the estimate by Falsey et al. before their additional corrections to account for seasonal variations in surveillance [3].

Our estimates suggest that the incidence of hospitalizations caused by RSV has been under-recorded among older adults. The substantial estimated incidence of hospitalizations attributable to RSV among those aged 65 and older agrees with earlier observations in a cohort study and time-series study, both of which showed that the incidence of hospitalizations attributable to RSV is heavily skewed toward older adults [17, 30]. This age group should be considered as a potential target population for RSV vaccines due to the potential high case-fatality risk after RSV infection [30, 31]. The number of hospitalizations recorded as being caused by RSV increased over the study period among patients 65 years of age and older. This trend may reflect changes in testing practices among older adults over time. To understand the actual incidence of hospitalizations attributable to RSV in the elderly, more frequent testing for RSV infections is needed.

Our results indicate that children in low-SES communities suffer from a particularly high incidence of RSV-associated hospitalizations. There are a number of potential causes for this disparity, including factors that might influence risk of viral infection, such as family size and the number of contacts; exposure to tobacco smoke and other pollutants; high prevalence of underlying respiratory diseases like asthma and chronic lung disease from prematurity; and shorter duration of breastfeeding [32, 33]. Additionally, decisions to admit patients could be influenced by the family’s SES and may vary based on factors such as the co-morbid illnesses of the patients, the reliability of follow-up, and the practices of individual clinicians.

Our results suggest that about 45% of hospitalizations for respiratory illnesses in infants < 1 year old are attributable to RSV infections. Monoclonal antibodies against RSV with an extended half-life [34], as well as vaccination of mothers and direct vaccination of infants using live-attenuated vaccines, might help to reduce the incidence of RSV infections [31].

There are several caveats to our results. First, we used hospitalizations due to RSV infections among children < 2 years of age as a proxy for the timing of RSV infections in the entire population; however, there may be differences in the timing of infections among the various age groups. In our preliminary analyses, we tested different time lags between the various age groups, but it did not improve our model fit. Since our model used monthly inpatient data, minor differences in timing between age groups are less likely to bias our analysis. Second, the cocirculation of other respiratory viruses may confound our estimates. We may overestimate hospitalizations attributable to RSV by not including infections due to respiratory viruses other than influenza and RSV as covariates. However, previous studies indicated that most other respiratory viruses do not have the same timing as epidemics of RSV infections [34, 35]. Therefore, the cocirculation of other respiratory viruses should have a relatively small impact on our estimates of RSV-attributable hospitalizations. Third, our estimates cannot separate RSV-bacterial coinfection from infection caused by RSV alone. Although pneumococcal pneumonia has been associated with RSV hospitalizations, pneumococcal pneumonia is unlikely to confound our estimates as pneumococcal infections are endemic, with similar incidence over time after accounting for vaccine-induced declines. Fourth, the estimates of recording ratios rely on the validity of estimates of the incidence of RSV-attributable hospitalizations. Since there is no gold standard for estimates of RSV-attributable hospitalizations, it is hard to validate our findings. Still, our age-based estimates of recording ratios are similar to those from a previous study conducted by the Centers for Disease Control and Prevention [17]. Lastly, we used individual-level hospital discharge data from 2005–2014 as state inpatient datasets normally lag several years. We stopped the analysis in 2014 because this was the year when coding shifted from ICD9 to ICD10. However, CDC sentinel surveillance suggests that RSV rates were stable over time before the COVID-19 pandemic [36]. Therefore, using more up-to-date data is unlikely to change our estimates.

In conclusion, children in families residing in low-SES areas had the highest incidence of RSV-associated hospitalizations for respiratory illnesses. The incidence of hospitalizations for RSV infections among older adults is greatly under-recorded. More comprehensive testing for RSV among older adults might help to better define the disease burden. Vaccines against RSV might provide substantial benefit to young children and older adults.

## Supplementary Information

**Additional file 1: Supplementary Methods.** **Table S1.** DIC scores of competing seasonality components. **Table S2.** ICD-9-CM-coded respiratory hospitalizations in the entire population and RSV hospitalizations among children <2 years in New Jersey, New York, and Washington. **Figure S1.** Estimated annual RSV-attributable respiratory hospitalization rates by age and SES group in New York, July 2005 - June 2014. **Figure S2.** Estimated annual RSV-attributable respiratory hospitalization rates by age and SES group in New Jersey, July 2005 - June 2014. **Figure S3.** Estimated annual RSV-attributable respiratory hospitalization rates by age and SES group in Washington, July 2005 - June 2014. 

## Data Availability

The hospitalization data are not available publicly but can be obtained from the State Inpatient Database upon signing a data use agreement with the Agency for Healthcare Research and Quality. The R code for this study can be found in the GitHub repository: https://github.com/weinbergerlab/RSVhospitalizations_USA.

## Abbreviations

RSV: Respiratory syncytial virus
SES: Socioeconomic status
US: The United States
DIC: Deviance information criterion
CrI: Credible interval
